# Attention-deficit hyperactivity disorder in children is related to maternal screen time during early childhood in Taiwan: a national prospective cohort study

**DOI:** 10.1186/s12888-023-05242-5

**Published:** 2023-10-10

**Authors:** Ping Shih, Tung-liang Chiang, Ping-I Lin, Ming-Yu Lin, Yue Leon Guo

**Affiliations:** 1 Department of Environmental and Occupational Medicine, National Taiwan University (NTU) and NTU Hospital, Taipei, Taiwan; 2 Department of Environmental and Occupational Medicine, National Taiwan University Hospital Hsin-Chu Branch, Hsinchu, Taiwan; 3 Institute of Health Policy and Management, College of Public Health, National Taiwan University, Taipei, Taiwan; 4 Discipline of Psychiatry and Mental Health, School of Clinical Medicine, University of New South Wales, Sydney, Australia; 5 Mental Health Research Unit, South Western Sydney Local Health District, Liverpool, Australia; 6 Department of Mental Health, School of Medicine, Western Sydney University, Penrith, Australia; 7 Department of Industrial Education, National Taiwan Normal University, Taipei, Taiwan; 8 Ministry of Education, Taipei, Taiwan; 9 National Institute of Environmental Health Sciences, National Health Research Institutes, Miaoli, Taiwan; 10 Institute of Environmental and Occupational Health Sciences, College of Public Health, National Taiwan University, Taipei, Taiwan; 11 Rm 339, 3F., No. 17, Xuzhou Rd., Zhongzheng Dist., Taipei City, 10055 Taiwan

**Keywords:** Screen time, Maternal exposure, ADHD, Preschool period, Primary caregiver

## Abstract

**Background:**

It is unclear to familial screen time in early childhood is associated with the subsequent diagnosis of attention-deficit and hyperactivity disorder (ADHD). Our study is to evaluate the association between screen time during early childhood in families and the incidence of ADHD.

**Methods:**

We conducted a population-based birth cohort study by using the Taiwan Birth Cohort Study, which recruited 24 200 mother–child pairs when children were 6 months old. Screen time exposure for children and parents were collected at the age of 18 and 36 months. Whether the child has ever been diagnosed with ADHD was determined at a follow-up interview at age 8. Factors including socioeconomic factors and screen time were analyzed using logistic regression to determine their association with the rate of ADHD.

**Results:**

A total of 16 651 term singletons were included in the final analysis. Of them, 382 (2.3%) were diagnosed as having ADHD before the age of 8 years. No significant relationship between children’s or fathers’ screen time and ADHD was noted. When compared to children whose mothers spent less time on screens, those whose mothers spent more than 3 h a day on screens when the child was 3 years old exhibited a higher incidence of ADHD (adjusted OR [aOR]: 1.31, 95% CI: 1.03–1.66).

**Conclusion:**

Higher maternal screen time when the child was 3 years old was associated with an increased incidence of ADHD in this population-based study. However, children’s screen time did not find related to ADHD. We found that it was the mother’s screen time, who typically serves as the primary caregiver in our study participants, not the child’s, that mattered. In addition to superficial screen use time, future research is needed to replicate the findings and clarify mechanisms underlying this association.

**Supplementary Information:**

The online version contains supplementary material available at 10.1186/s12888-023-05242-5.

## Introduction

The *Diagnostic and Statistical Manual of Mental Disorders, Fifth Edition* (*DSM-5*) introduced a new diagnostic category termed neurodevelopmental disorders (NDDs) in 2013 [1]. Among NDDs, attention-deficit hyperactivity disorder (ADHD) is the most common disorder. Individuals with ADHD display a consistent pattern of inattention and/or hyperactivity-impulsivity, including difficulty paying attention, avoiding tasks that demand sustained mental effort, restlessness, excessive talking, and an inability to remain seated. The prevalence of ADHD diagnosed on the basis of *DSM-5* criteria was 5–11% in individuals aged < 18 years [2]. The National Survey of Children’s Health indicates that the prevalence of parent-reported ADHD in children aged from 4 to 17 years in the United States increased from 7.8% to 2003 to 11% in 2011 [3]. In Taiwan, the prevalence of ADHD in junior high school students was 7.5% in 2005 [4]. A national survey conducted during 2015 to 2016 in children aged 9 to 13 years indicated that the weighted lifetime prevalence rate of ADHD was10.5% [5]. Symptoms of ADHD often continue to persist into adulthood and pose a substantial burden on patients, families, and society [6]. This burden encompasses emotional and social impairment, accidental injuries, involvement in criminal activities and delinquency, as well as educational underachievement. The increasing prevalence of ADHD not only underscores the importance of early detection and intervention but also imply the role of environmental factors in susceptibility to ADHD.

Emerging evidence suggests a relationship between screen time and ADHD symptoms. Using a US national longitudinal sample, a study published in 2004 determined the association between early television viewing at the ages of 1 and 3 years and subsequent inattention at the age of 7 years [7]. However, a study published some years later that used the same cohort data did not determine a detrimental effect of screen time on attention [8]. A meta-analysis including related studies published until September 2013 demonstrated a marginally significant relationship between media use and ADHD-related behaviors despite the heterogeneity of the studies [9]. Moreover, a recent systematic review and meta-analysis identified a weak but significant association between screen time and children’s internalizing and externalizing (aggression or ADHD symptoms) behaviors [10]. In 2019, the World Health Organization (WHO) published guidelines indicating that children aged from 2 to 4 years should have no more than 1 h of screen time [11]. Furthermore, the American Academy of Pediatrics (APA) recommends that the screen time of children aged from 2 to 5 years should not exceed more than 1 h per day [12]. Warnings have been issued regarding the adverse effects of screen exposure on children’s health.

Although studies have demonstrated the association between screen time and ADHD development, the role of screen time in ADHD remains elusive due to the presence of heterogeneous evidence across different studies. Furthermore, parenting should not be overlooked when evaluating the effects of media use in early childhood, especially when media can serve as a substitute for parent–child interactions [13]. Kids in Taiwan: National Longitudinal Study of Child Development and Care (KIT) indicated that the average screen time of 3-year-old children was 2 h and 17 min per day; these screen times are still longer than those recommended by the WHO and APA [14]. Therefore, understanding the association between preschool screen exposure and children’s health can provide insights into public health and clinical practices related to ADHD.

To the best of our knowledge, no study had evaluated the association between screen time and ADHD by using a Taiwanese population sample. Therefore, this study examined the association between screen exposure in not only children but also parents and ADHD development during early childhood by using a representative birth cohort sample in Taiwan.

## Methods

### Study Population

**Fig. 1:**
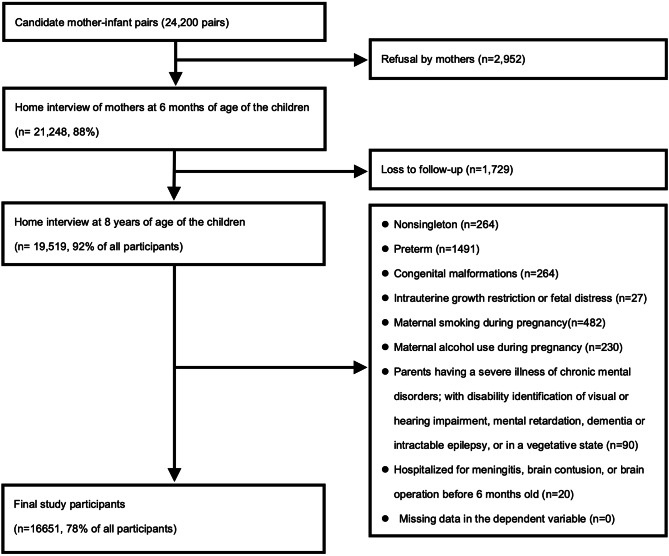
Flowchart of data collection in the study

The Taiwan Birth Cohort Study (TBCS) is a national prospective longitudinal cohort study. In this study, a 2-stage stratified random sampling method was used to select infants born in 2005 in Taiwan from the National Birth Report database. We included 24 200 representative mother–infant pairs, which represented approximately 12% of all births in Taiwan in 2005. The details are described previously [15]. Structured questionnaires were administered by well-trained interviewers when children were aged 6 months, 18 months, 3 years, and 8 years. The exclusion criteria were as follows: being lost to follow-up, having multiple gestations, having preterm birth, congenital malformations, experiencing intrauterine growth restriction or fetal distress, smoking and consuming alcohol during pregnancy, being hospitalized for meningitis, and having a brain contusion or brain surgery before the age of 6 months. Furthermore, we excluded parents with a disability certificate for chronic mental health conditions, autism spectrum disorder, visual or hearing impairment, mental retardation, dementia, and intractable epilepsy; those in a vegetative state; and those with severe chronic mental disorders. Finally, we included 16 651 mother–infant pairs (Fig. 1).

### Variables

Several interviews were conducted when children were aged 6 months, 18 months, 3 years, and 8 years. We collected information on maternal conditions during pregnancy, infant birth outcomes, child health conditions, parental socioeconomic status, prenatal traffic-related air pollution [16], and other demographic factors when the first-wave of survey. Parents’ exposure was determined on the basis of self-reported average daily screen time when the child was aged 18 months and 3 years. During the interview, parents answered open-ended questions on how many hours and minutes they spent watching television, videotapes, DVDs, and computers, excluding the screen time during work. A child’s screen time was determined on the basis of the primary caregiver’s response to the following question: How much time does your baby watch television (including videotapes, DVDs) on average each day? High exposure was considered when the time spent exceeded the 67th percentile in relation to the screen time of the entire sample.

The status of parent-reported community diagnoses of ADHD was determined using questionnaires administered by trained interviewers. The response “yes” from the main caregiver was selected when ADHD was diagnosed by a physician, psychologist, or special educator by the age of 8 years.

Covariates that were adjusted for included the child’s sex, maternal age at childbirth, maternal education level, urban living, household income, marital relationship, family supports after childbirth, maternal unintentional pregnancy and postpartum smoking, including secondhand smoke [17, 18]. Information on these potential confounders was collected through face-to-face interviews during the first survey when children were aged 6 months. To be more precise, mother’s highest level of education was recorded. An illiterate person is considered as an uneducated person. The calculation encompasses the 1st to the 6th year of primary school, with junior high school education being coded as 7 to 9 years of education, high school or vocational school as 10 to 12 years, and any education beyond the 12th year is categorized as college or graduate school, based on the total years of education completed. Residence was determined by whether the baby’s current living area was in an urban or non-urban location, which included township streets, villages, or rural areas. Household income data were collected through the question: “What was the average monthly income for your household over the past year?“ Marital relationships were classified based on the current marital status of the baby’s parents. If the response indicated “Married, living together as husband and wife,“ it was categorized as a married status. All other responses, such as divorced, deceased, or single, were grouped together as “others.“ In terms of family support, when facing challenges, the usual availability of effective assistance from family members is indicative of good family support. Unintentional pregnancy raised from the question: was the conception of this baby planned, or was it the result of an unforeseen pregnancy?

Additionally, primary daytime and nighttime caregivers were surveyed, and their responses were combined to identify the caregivers responsible for the children throughout the day when they were 18 months and 36 months old. The frequency of exposure to violent screen content, including scenes of fighting and quarrels, as well as the consumption of pornographic and sexual content, was documented by the main caregiver when the children were 36 years old.

### Statistical analysis

The binary outcome variable was the parent-reported community diagnosis of ADHD in children, and the main predictors were children’s and parents’ screen time in early childhood. The relationship between children’s and parents’ screen time was estimated using the Spearman rank-based correlation test. Furthermore, a logistic regression model was used to determine the association of children’s and parents’ screen time when the children were aged 18 months and 3 years with the rate of ADHD after adjustment for confounders. Furthermore, the relationships between media content and the subsequent incidence of ADHD were also assessed. The findings are reported as the adjusted odds ratio (aOR) and 95% confidence interval (CI) for the occurrence of ADHD. Statistical analyses were performed using JMP (version 16, SAS Institute Inc., USA). A 2-sided P value of < 0.05 indicated statistical significance.

## Results

Among the 16 651 mother–infant pairs included in the final analysis (Fig. 1), 2.3% of children were diagnosed as having ADHD by the age of 8 years. Slightly more than half of the children were boys, and 10% of mothers of these children were aged ≥ 35 years at childbirth. Slightly less than half of the mothers had a high school education or above or had been exposed to smoking or secondhand smoke during postpartum period, and less than half of the participants lived in urban areas, or had a annual household income of USD 20 000 or less. Most of these parents lived together and about one fourth were unintentional pregnancy. Table 1 summarizes the demographic characteristics of the children with or without ADHD.

**Table 1:** Demographic characteristics of the study population

Covariates	N = 16,651	ADHD	Non-ADHD	P-value
	No· (%)	382 (2·3%)	16,245 (97·7%)	
Gender of the child				<·0001
Boy	8691 (52·2%)	311 (3·6%)	8380 (96·4%)	
Girl	7960 (47·8%)	71 (0·9%)	7889 (99·1%)	
Maternal age at childbirth				0·0120
< 35 years old	14,969 (90·0%)	329 (2·2%)	14,640 (97·8%)	
≥ 35 years old	1673 (10·1%)	53 (3·2%)	1620 (96·8%)	
Maternal education				<·0001
Junior high school or lower	8865 (53·3%)	245 (2·8%)	8620 (97·2%)	
Senior high school or above	7766 (46·7%)	135 (1·7%)	7631 (98·3%)	
Annual household income				0·0134
≤ 20,000 USD	6865 (41·2%)	181 (2·6%)	6684 (97·4%)	
> 20,000 USD	9786 (58·8%)	201 (2·1%)	9585 (98·0%)	
Residence				0·0122
Urban area	7827 (47·1%)	204 (2·6%)	7623 (97·4%)	
Non-urban area	8800 (52·9%)	178 (2·0%)	8622 (98·0%)	
Marital relationship				
Married	15,816 (95·0%)	348 (2·2%)	15,468 (97·8%)	0·0004
Divorced/separated/single	835 (5·0%)	34 (4·1%)	801 (95·9%)	
Family supports after childbirth				
Good	10,604 (63·9%)	213 (2·0%)	10,391 (98·0%)	0·0019
Poor	5982 (36·1%)	165 (2·8%)	5817 (97·2%)	
Maternal unintentional pregnancy				
Yes	4649 (27·9%)	135 (2·9%)	4514 (97·1%)	0·0011
No	12,002 (72·1%)	247 (2·1%)	11,755 (97·9%)	
Postpartum smoking (including secondhand smoke)				
Yes	6779 (40·7%)	170 (2·5%)	6609 (97·5%)	0·1279
No	9869 (59·3%)	212 (2·2%)	9657 (97·9%)	

The *p* values were derived from a chi-square test

Table 2 presents the distribution of children’s and parents’ screen time when the children were aged 18 months and 3 years. The mean screen time was more than 100 min a day, which is higher than the recommendation of no more than 1 h of screen time for children aged 2 to 5 years according to the WHO and APA. We noted a significant correlation between children’s and parents’ screen time in the different periods of early childhood (Table 3).

**Table 2:** Distribution of screen time in children aged 18 months and 3 years

Screen time (minutes)	Mean ± SD	IQR	Minimum	First quartile	Median	Third quartile	Maximum
At 18 months of age							
Mother	160·54 ± 112·54	150	0	60	120	210	900
Father	138·68 ± 88·89	120	0	60	120	180	900
Child	104·7 ± 108·54	90	0	30	60	120	780
At 3 years of age							
Mother	154·51 ± 106·86	120	0	60	120	180	1080
Father	151·4 ± 93·59	90	0	90	120	180	960
Child	161·86 ± 110·94	180	0	60	120	240	840

IQR, interquartile range; SD, standard deviation·

**Table 3:** Correlation between the screen time of children and parents

Screen time	At 18 months of age			At 3 years of age		
	Mother	Father	Child	Mother	Father	Child
At 18 months of age						
Mother	1					
Father	0·4751***	1				
Child	0·2465***	0·1707***	1			
At 3 years of age						
Mother	0·5578***	0·305***	0·1680***	1		
Father	0·2883***	0·4329***	0·1162***	0·4667***	1	
Child	0·2778***	0·2010***	0·3353***	0·3668***	0·2700***	1

The correlations were estimated by Spearman rank-based correlation test. *** *p* value < 0·001

In our study group, when the child is about 18 months old, most of the children were mainly cared for by the mother: mother with the help of relatives (43.6%), the mother alone (36.2%), the parents themselves (2.1%), and about 18% of the children were mainly raised by relatives, including grandparents. Furthermore, at about 36 months, 28.8% of the children were mainly taken care of by the mother alone, 23.8% were cared for by the mother and relatives, 20.6% went to kindergarten or nurseries during the day, and 12.1% were raised mainly by relatives. Only 6.7% of the participants were primarily cared for with the help of babysitters (Supplementary Table 1).

Table 4 presents the results of logistic regression performed to determine the relationship between ADHD and screen time during early childhood. A significant association was noted between ADHD diagnosis by the age of 8 years and maternal screen time when the child’s age was 18 months and 3 years. However, no association of children’s or fathers’ screen time with the rate of ADHD in children was noted. After adjustment for child’s sex, maternal education, maternal age at childbirth, urban living, family income, marital relationship, family supports, maternal unintentional pregnancy and postpartum smoking (including secondhand smoke), prenatal ambient nitrogen oxides concentration and temperature, mothers’ screen time when children were aged 3 years was significantly related to ADHD diagnosis (aOR: 1.31, 95% CI: 1.03–1.66). A subgroup analysis was conducted to examine differences between boys and girls, between high-income and low-income families, and between families living in urban and nonurban areas. The effect of maternal screen time when children were aged 3 years was observed on boys and low-income families (Supplementary Table 2). When examining the content of media exposure, children who were frequently or occasionally exposed to violent content at the age of 3 had a higher rate of developing subsequent ADHD compared to those who were never exposed (Supplementary Table 3).

**Table 4:** Association between screen time at the age of 18 months and 3 years and ADHD

Variables	No. (%)	ADHD (n = 382, 2·3%)	
		OR (95% CI)	aOR [1] (95% CI)
At 18 months of age			
Mother			
> 67th (longer than 3 H)	4299 (26·7%)	1·28 (1·02 − 1·59)*	1·22 (0·97 − 1·54)
≤ 67th (shorter than 3 H)	11,783 (73·3%)	1	1
Father			
> 67th (longer than 3 H)	3008 (18·8%)	1·21 (0·94 − 1·56)	1·20 (0·93 − 1·56)
≤ 67th (shorter than 3 H)	12,985 (81·2%)	1	1
Child			
> 67th (longer than 2 H)	4028 (24·9%)	1·22 (0·97 − 1·53)	1·18 (0·93 − 1·49)
≤ 67th (shorter than 2 H)	12,149 (75·1%)	1	1
At 3 years of age			
Mother			
> 67th (longer than 3 H)	3807 (24·1%)	1·36 (1·08 − 1·72)**	1·31 (1·03 − 1·66)*
≤ 67th (shorter than 3 H)	11,991 (75·9%)	1	1
Father			
> 67th (longer than 3 H)	3553 (22·6%)	1·23 (0·97 − 1·58)	1·19 (0·92 − 1·52)
≤ 67th (shorter than 3 H)	12,173 (77·4%)	1	1
Child			
> 67th (longer than 3 H)	4530 (28·1%)	1·14 (0·91 − 1·43)	1·09 (0·86 − 1·37)
≤ 67th (shorter than 3 H)	11,605 (71·9%)	1	1

Model 1 was adjusted for child’s sex, urban living, maternal age at childbirth, maternal education level, household income, marital relationship, family supports after childbirth, maternal unintentional pregnancy and postpartum smoking (including secondhand smoke), prenatal ambient NOx, and temperature

* *p* value < 0·05; ** *p* value < 0·01; H, hour; CI, Confidence interval; OR, odds ratio; aOR, adjusted odds ratio

## Discussion

Our results revealed that mothers’ screen time when children were aged 3 years was positively associated with the incidence of ADHD in children by the age of 8 years. Notably, the role of children’s and fathers’ screen time in the rate of ADHD during early childhood appears to be limited.

In our study, we observed a parent-reported community diagnoses of ADHD prevalence of 2.3% in 8-year-old participants in 2013. This prevalence is considerably lower than that of 6.3% in students aged 6 to 9 years reported in a field study conducted by a group including psychiatrists [19]. The low prevalence rates observed in this study, as determined through community diagnoses, may potentially be lower than epidemiological estimates derived from comprehensive mental health assessments, indicating a potential underdiagnosis However, our observed prevalence is similar to that reported in a study using Taiwan’s National Health Insurance Research Database (NHIRD), which covered 99.9% population in Taiwan; this study indicated that 2.23% of children aged from 7 to 12 years underwent medical assessment and treatment for ADHD in 2011 [20]. Moreover, according to the NHIRD, the prevalence of ADHD in male youth was approximately 4 times higher than that in female youth; this finding is consistent with that of our study population. Thus, children with ADHD in our study may reasonably represent those diagnosed with ADHD in medical settings.

Screen time refers to the time spent watching television, playing a video game, or using an electronic device with a screen (such as a computer, smartphone or tablet). The WHO defines it as “time spent passively watching screen-based entertainment (television, computer, and mobile devices), and it does not include active screen-based games where physical activity or movement is required.” During 2005 to 2008 when the TBCS was conducted and data on screen time were collected, the research sample was less than 3 years old. Taiwanese families then did not use mobile electronic devices, such as smartphones and tablets, on a daily basis. Although the iPhone was first launched in 2007, it was not officially available in Taiwan for purchase until the end of 2008. The iPad was launched in 2010. Therefore, at that time, toddlers younger than 3 years mainly watched television, videotapes, DVDs, and some computers. The average exposure time was 1 h and 45 min in 18-month-old children and 2 h 42 min in 3-year-old children. This screen time of 3-year-old children is similar to that of more than 2 h per day, including computers, communication, and consumer electronics, determined from KIT in 2013 to 2014 [14]. The global average prevalence in children aged 2 to 5 years who met screen time guidelines (1 h per day) was 35.6% [21], and in our participants, it was approximately one-quarter. A positive correlation was noted between children’s and parents’ screen time, which is consistent with the findings of a previous study in preschool children [22]. In recent years, with changes in the social atmosphere, the habit of using screen electronic devices has become increasingly common. In addition, during the COVID-19 pandemic, an estimated 66 more minutes of screen time per day have increased among preschoolers aged less 5 years and an additional hour per day among adults aged over 18 years [23, 24]. Further research is warranted to determine the effects of such changes on health.

Studies have reported a relationship between screen time, including television viewing, game playing, video watching, and computer using, and ADHD symptoms or behaviors and externalizing behaviors [9, 10, 25]. In Taiwan, prolonged touch screen device usage was found to be associated with attention problems and aggressive behaviors in toddlers aged from 18 to 36 months [26]. However, despite the increasing number of cohort studies focusing on the relationship between preschool exposure to screen and the incidence of ADHD diagnosis, this association remains inconclusive. A Dutch cohort study determined that screen time, with an average of 29.4 min per day for 2-year-old children and 58.3 min per day for 4- to 5-year-old children, was cross-sectionally related to externalizing symptoms during early childhood but not associated with the diagnosis of ADHD at the age of 8 to 10 years [27]. The possible explanation is that screen exposure affects short-term behaviors without exerting sustaining effects. In addition, variations in the amount, frequency, content, and types of media of screen exposure may account for the elusive long-term adverse health effects. Few studies have examined the effects of children’s screen time on ADHD while considering mothers’ screen time. A study on German children using a cross-sectional approach determined that higher maternal screen time was significantly associated with children’s higher screen time, and both were associated with ADHD symptoms among 2- to 9-year-old children [28]. Our findings partially fail to indicate the association between children’s screen time and ADHD risk, which may be attributable to differences in the study methodology and the definition of dependent variables. Because of the small number of studies and inconsistent evidence, more research is warranted.

The Differential Susceptibility to Media Effects Model (DSMM) is a theory in media studies that suggests individuals vary in their susceptibility or responsiveness to media messages and content [29]. This model posits that people have different levels of vulnerability to media influences based on factors such as their personality traits, demographics, and life experiences. Some individuals may be more susceptible to the effects of media, while others are less so. The DSMM can also theoretically interpret the effect of media usage on ADHD-related behaviors [30]. The most explanatory hypotheses state that children’s media-evoked responses following the fast pace or violent nature of media increase the likelihood of ADHD-related behaviors. To confirm this, we did exam the content of media. Exposing children to violent but not erotic content may also be linked to further ADHD diagnoses. (Supplementary Table 3) In terms of developmental and dispositional susceptibility, we did evaluate media exposure among young children and investigated sex differences in susceptibility, showing significant outcomes for mothers’ screen exposure among boys (Supplementary Table 2).

We assumed that the adverse effect of longer exposure among mothers during early childhood fits the social susceptibility hypothesis in the DSMM, such as parenting style and media-specific parenting. As demonstrated in Supplementary Table 2, mothers were the most commonly identified primary caregivers among the study participants. In addition, we had information on how often mothers watch TV with their children at the age of 36 months. However, all answers were “always” and no further information was given. Parenting style is a factor for ADHD-related behaviors [31]. Parenting style, demographic factors, and parental well-being amplify the effects of children’s media use on ADHD-related behaviors [32, 33]. A study examining the effects of television on the interaction between parents and children aged under 3 years determined that when the television was on, the quality and quantity of parent–child interaction decreased compared with when it was off [34]. In 2009 in Taiwan, one of the family pastimes that preschool students participated in the most was watching television, including videotapes, although it does not contribute to the establishment of parent–child interaction [35, 36]. Nevertheless, a German study reported that children’s and mothers’ media use and parent–child interaction independently contribute to ADHD behaviors [28]. The correlation of screen time between the two is expected because when one person is watching a television or videotape, the other is also watching. Thus, parent-reported screen time may underestimate the screen time of some children who watched TV along with their parents. Furthermore, the amount of screen time may reflect parenting styles, parent–child interaction, or other intrinsic biopsychosocial factors in parents instead of environmental exposure per se. This study found that the lack of significant correlation between paternal screen time and ADHD diagnosis, which could also be interpreted since the majority of children were cared for by mothers. We believe that the manner or method of companionship is very important, because some companionship may just be with them without any guidance or education, and some are not. However, we cannot precisely break down the total time watched together or individually, and it would be inappropriate to overinterpret the current information. The content of the media and why and how these electronic devices are used are crucial. Thus, the current evidence should be confirmed in future studies on the long-term effects of screen time on ADHD.

The strength of this study is that it provides new insights into the relationship between early childhood screen time among children and mothers and ADHD onset. The study benefited from a representative sample of Taiwanese infants born in 2005 with a population-based cohort design that responded well to detailed data on covariates. Furthermore, the independent variable was collected when children were aged 18 and 36 months, which minimized recall bias for the target dependent disease or symptom collected years later. A limitation of the current study is that ADHD and screen time were assessed on the basis of parental reports, which may have contributed to reporting bias. The prevalence of this diagnosis is similar to that in the same age group in Taiwan’s NHIRD. Thus, our sample of children with ADHD is likely representative of the community-based population. That said, self-reactive overdiagnosis was unlikely to occur in our study sample. Despite concerns regarding the underdiagnosis of ADHD, ADHD diagnosed by the age of 8 years might reasonably represent a proportion of more severe cases, and some children with ADHD may be observed in subsequent years. The study may be, therefore, biased toward children with more severe ADHD symptoms. Moreover, exact data on the amount of screen time and the time children spent gazing at the screen were not available. Information on pattern, such as continuous exposure and interval rest periods, or parenting factors while watching television/videotapes were not available in our dataset. Thus, the variation in these parameters cannot be explored in the current study. Obtaining such information would have been impractical in a large cohort study conducted more than 10 years ago. People are likely to underestimate the amount of TV they or their children watch, especially those with more screen time. In theory, this misclassification might reduce the observed association. Nonetheless, the observation of a significant relationship between maternal screen time and ADHD diagnoses suggests that the actual effect may be stronger. Additionally, 8% of survey participants were not followed up when their child was approximately 8 years old and did not provide a reason for the refusal. Therefore, whether rejection is associated with clinical features related to ADHD remains unclear. Overall, the impact of bias is acceptable because of the high participation rate.

Nowadays, we are expecting to see improvements in measurement and inspection equipment to more accurately assess screen viewing, for example, using portable devices and identifying the wavelength and brightness of screen light sources. Besides, different forms of screens, content, whether there is parental guidance to watch, watching TV alone or with adults, etc. are all aspects worthy of further research and exploration. Moreover, the causes and mechanisms behind screen time that cause health problems need to be further clarified. Nevertheless, long-term health effects, dosage effects, recommendations for appropriately limiting screen time or duration per session, appropriate timing of viewing (e.g. while eating, waiting, in bed) all require research for further clinical application. Last but not the least, with the proliferation of smartphones, tablets, and other multifunctional devices in our daily lives, detailed information on screen exposure should be captured in the near future.

## Conclusions

The current study suggests that increased maternal screen time when the child was 3 years old was associated with an increased incidence of ADHD in children by the age of 8 years. Evidence of a link between a child’s screen time during early childhood and the later diagnosis of ADHD was not observed. The findings may suggest a connection between the screen time of the primary caregiver and subsequent ADHD diagnosis, given that the primary caregiver is typically the mother in our sample. Further research with more detailed measurements for screen time is warranted to clarify these findings.

### Electronic supplementary material

Below is the link to the electronic supplementary material.

Supplementary Material 1

Supplementary Material 2

Supplementary Material 3

## Data Availability

The data are available and can be accessed by application to the Health and Welfare Data Science Center, Ministry of Health and Welfare, at https://dep.mohw.gov.tw/dos/np-2497-113.html.

## List of Abbreviations

DSM-5: Diagnostic and Statistical Manual of Mental Disorders, Fifth Edition
NDDs: Neurodevelopmental disorders
ADHD: Attention-deficit hyperactivity disorder
WHO: World Health Organization
APA: American Academy of Pediatrics
KIT: Kids in Taiwan: National Longitudinal Study of Child Development and Care
TBCS: Taiwan Birth Cohort Study
DSMM: Differential Susceptibility to Media Effects Model
NHIRD: National Health Insurance Research Database
