# Supplementary material for: Attention-deficit hyperactivity disorder in children is related to maternal screen time during early childhood in Taiwan: a national prospective cohort study
Source: BMC Psychiatry. 2023 Oct 10;23:736. doi: 10.1186/s12888-023-05242-5 (PMC10565960; doi:10.1186/s12888-023-05242-5)
Supplement: Supplementary file 1 — Supplementary Material 1 [file 12888_2023_5242_MOESM1_ESM.docx]

**Supplementary Table 1.** **Primary caregiver when children were aged 18 months and 3 years old.**

| **Primary caregiver** | N = 16,651 |
| --- | --- |
|  | No· (%) |
| At 18 months of age |  |
| Mother and relatives | 6382 (38·3%) |
| Mother alone | 6034 (36·2%) |
| Relatives | 2988 (18·0%) |
| Parents and relatives | 882 (5·3%) |
| Parents | 346 (2·1%) |
| Father alone | 18 (0·1%) |
| others | 1 (0·0%) |
| At 3 years of age |  |
| Mother alone | 4799 (28·8%) |
| Mother and relatives | 3958 (23·8%) |
| Kindergartens/Nurseries/Child Care Centers in the daytime | 3429 (20·6%) |
| *In the nighttime* |  |
| *Mother alone* | *2701 (16*·*2%)* |
| *Parents* | *280 (1*·*7%)* |
| *Relatives* | *172 (1*·*0%)* |
| *Mother and relatives* | *167 (1*·*0%)* |
| *Father alone* | *58 (0*·*4%)* |
| *Others* | *51 (0*·*3%)* |
| Relatives | 2021 (12·1%) |
| With help of babysitter | 1120 (6·7%) |
| Parents and relatives | 881 (5·3%) |
| Parents | 418 (2·5%) |
| Father alone | 23 (0·1%) |
| Others | 2 (0·0%) |
