# Supplementary material for: Attention-deficit hyperactivity disorder in children is related to maternal screen time during early childhood in Taiwan: a national prospective cohort study
Source: BMC Psychiatry. 2023 Oct 10;23:736. doi: 10.1186/s12888-023-05242-5 (PMC10565960; doi:10.1186/s12888-023-05242-5)
Supplement: Supplementary file 2 — Supplementary Material 2 [file 12888_2023_5242_MOESM2_ESM.docx]

**Supplementary Table 2.** **Sensitivity analysis for the association between maternal screen time when children were aged 3 years and ADHD diagnosis by the age of 8 years, stratified by child’s sex, family income, and residency.**

| Girl  Boy | 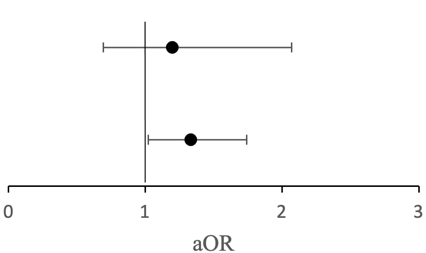   \| 1·20 (0·69 - 2·07) \| \| --- \|  \| 1·33 (1·02 - 1·74)* \| \| --- \| |
| --- | --- | --- | --- |
| High-income family  Low-income family | \| 1·05 (0·72 - 1·54) \| \| --- \| \|  \| \| 1·53 (1·11 - 2·11)** \|   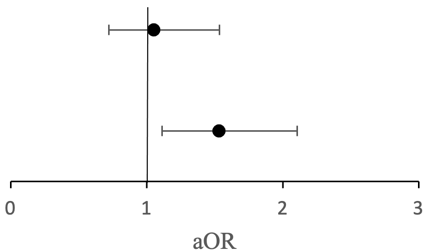 |
| Non-urban living  Urban living | 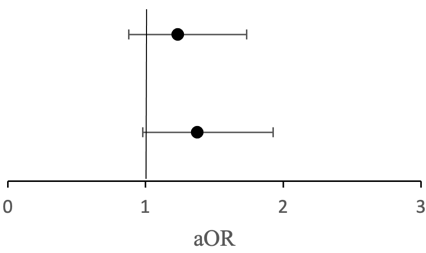   \| 1·23 (0·88 - 1·74) \| \| --- \| \|  \| \| 1·37 (0·98 - 1·93) \| |

The model is equivalent to model 1 in Table 4, excluding stratification factors. The association was demonstrated in the form of adjusted odds ratio (95% confidence interval) · *P < 0·05; **P < 0·01

A low annual household income was defined as ≤ $20,000 per year, while a high income was defined as >$20,000.
