# Supplementary material for: Attention-deficit hyperactivity disorder in children is related to maternal screen time during early childhood in Taiwan: a national prospective cohort study
Source: BMC Psychiatry. 2023 Oct 10;23:736. doi: 10.1186/s12888-023-05242-5 (PMC10565960; doi:10.1186/s12888-023-05242-5)
Supplement: Supplementary file 3 — Supplementary Material 3 [file 12888_2023_5242_MOESM3_ESM.docx]

**Supplementary Table 3. Association with ever exposing screen containing violent and pornographic content and subsequent onset of ADHD.**

| Variables | No· (%) | ADHD | Non-ADHD | OR (95% CI) | aOR (95% CI) |
| --- | --- | --- | --- | --- | --- |
|  |  | 382 (2·3%) | 16245 (97·7%) |  |  |
| Violent content | |  |  |  |  |
| Frequent or sometimes | 2226 (13·8%) | 66 (3·0%) | 2160 (97·0%) | 1·52 (1·13 - 2·05)** | 1·36 (1·00 - 1·85)* |
| Scarcely | 6948 (43·1%) | 162 (2·3%) | 6786 (97·7%) | 1·19 (0·95 - 1·50) | 1·16 (0·92 - 1·47) |
| Never | 6961 (43·1%) | 345 (2·2%) | 15194 (97·8%) | 1 | 1 |
| Pornographic content | |  |  |  |  |
| Ever | 597 (3·7%) | 228 (2·5%) | 8946 (97·5%) | 1·53 (0·965 - 2·414) | 1·38 (0·86 - 2·22) |
| Never | 15539 (96·3%) | 137 (2·0%) | 6824 (98·0%) | 1 | 1 |

Note:

1. Ever exposing screen containing violent and pornographic content collected when the child was 3 years old.
2. The model is equivalent to model 1 in Table 4, adding one more factor related to media content. aOR, adjusted odds ratio· *P < 0·05, **P < 0·01
3. Taking into account children's exposure to violent television content and mothers' screen time at age 3, the analysis still showed that the association between maternal screen time and ADHD remained significant.
